# Kinetics of Highly Sensitive Troponin T after Cardiac Surgery

**DOI:** 10.1155/2015/574546

**Published:** 2015-10-11

**Authors:** Amr S. Omar, Suraj Sudarsanan, Samy Hanoura, Hany Osman, Praveen C. Sivadasan, Yasser Shouman, Alejandro Kohn Tuli, Rajvir Singh, Abdulaziz Al Khulaifi

**Affiliations:** ^1^Department of Cardiothoracic Surgery/Cardiac Anaesthesia & ICU Section, Heart Hospital, Hamad Medical Corporation, P.O. Box 3050, Doha, Qatar; ^2^Department of Critical Care Medicine, Beni Suef University, Beni-Suef 62511, Egypt; ^3^Department of Anesthesia, Al-Azhar University, Cairo 11651, Egypt; ^4^Department of Medical Research, Hamad Medical Corporation, P.O. Box 3050, Doha, Qatar

## Abstract

Perioperative myocardial infarction (PMI) confers a considerable risk in cardiac surgery settings; finding the ideal biomarker seems to be an ideal goal. Our aim was to assess the diagnostic accuracy of highly sensitive troponin T (hsTnT) in cardiac surgery settings and to define a diagnostic level for PMI diagnosis. This was a single-center prospective observational study analyzing data from all patients who underwent cardiac surgeries. The primary outcome was the diagnosis of PMI through a specific level. The secondary outcome measures were the lengths of mechanical ventilation (LOV), stay in the intensive care unit (LOSICU), and hospitalization. Based on the third universal definition of PMI, patients were divided into two groups: no PMI (Group I) and PMI (Group II). Data from 413 patients were analyzed. Nine patients fulfilled the diagnostic criteria of PMI, while 41 patients were identified with a 5-fold increase in their CK-MB (≥120 U/L). Using ROC analysis, a hsTnT level of 3,466 ng/L or above showed 90% sensitivity and 90% specificity for the diagnosis of PMI. Secondary outcome measures in patients with PMI were significantly prolonged. In conclusion, the hsTnT levels detected here paralleled those of CK-MB and a cut-off level of 3466 ng/L could be diagnostic of PMI.

## 1. Introduction

Biomarkers are important diagnostic tools for addressing clinical problems. Recent changes in laboratory diagnostic power have resulted in these markers being incorporated into international guidelines and into the updated definition of myocardial infarction [1]. An ideal biomarker for myocardial infarction diagnosis should possess high sensitivity and specificity, also rapidly released and slowly eliminated, for early and late diagnoses. In addition, such a biomarker should be cost-effective and simple to use, without affecting patients' outcome or impacting their therapy [1]. Three subunits (C, I, and T) located on the myofibrillar thin (actin) filament of striated (skeletal and cardiac) muscle shape the backbone of the troponin complex. Cardiac muscle expresses the troponin T and I isoforms, so cardiac troponin T (cTnT) and I (cTnI) are more specific than creatine kinase (CK) values for myocardial injury and, owing to their high sensitivity, may rise when creatine kinase MB (CK-MB) concentrations do not [2].

Many contributors may raise cardiac enzyme levels after cardiac surgery, such as acute coronary syndrome (ACS) related to recent myocardial infarction (AMI) before the surgery [3], or directly related to cardiac surgery in perioperative myocardial infarction (PMI), such as inadequate cardiac protection, reperfusion injury, and direct surgical trauma [4]. Such elevations during cardiac surgery may not be ACS-related, as these enzymes may be already elevated in patients with end-stage renal disease [5], acute pericarditis, acute heart failure (AHF) [6], sepsis [7], or rhabdomyolysis [8].

The PMI could not be solely attributed to coronary surgery (whether on- or off-pump), as it might also be associated with isolated valvular surgeries, although this association is not common [9, 10]; McGregor et al. reported an incidence of 4% with valvular surgeries [10].

Highly sensitive troponin T (hsTnT) is a reliable biomarker with high sensitivity and negative predictive values compared with conventional troponin (cTn) [11]. For the diagnosis of acute myocardial infarction (AMI), hsTnT offers excellent diagnostic performance even with early presentation to the emergency department [12] and some evidence exists for a better diagnostic accuracy than cTn [13].

Open heart surgeries carry a well-established risk of PMI [14]; 2–5% incidence had been reported with intense mortality and morbidity [15]. Since the prognosis of patients after cardiac surgery must also be addressed, a marker with predictive power in both short- and long-term mortality should be optimum. In a meta-analysis by Lurati Buse et al., the authors studied the prognostic value of cTn in cardiac surgery settings, where postoperative cTn release was found to be associated with mid- and short-term all-cause mortality (12 mo and 30 d, resp.) [16]. The universal diagnosis of PMI is based on an increase in the CK-MB by more than 5 times the 99th percentile upper reference level plus either new pathological Q-waves or left bundle branch block (LBBB) on 12 lead electrocardiogram (ECG), image evidence of new viable myocardium loss, or angiographic findings of native coronary occlusion or new graft occlusion [17]. The role of hsTnT in the diagnosis of PMI has not yet been defined.

## 2. Aim of the Study

The aim of the study is to define the role of hsTnT in diagnosing PMI in the cardiac surgery setting.

## 3. Methods

### 3.1. Study Design

This is a single-center prospective observational study conducted over a 2-year period (October 2011 to September 2013) in a 12-bed cardiothoracic ICU of a Qatari heart hospital (Hamad Medical Corporation, Doha, Qatar). The ethical committee gave the approval to conduct the study (reference number 13223/13), with a waiver of informed consent, as no specific intervention was performed and no extra blood sampling was required. Patients with chronic renal impairment, sepsis, or a preexisting high level of hsTnT were excluded (unless postoperative difference is significant by more than a 50% increment). Patients with marked intraoperative hypotension (mean arterial blood pressure less than 80 for more than 5 min) were also excluded (32 patients).

We enrolled 413 patients who underwent cardiac surgeries. Based on the association of PMI, patients were divided into two groups (Tables 3 and 4). According to their hsTnT levels (corresponding to a 5-fold increase in CK-MB), patients were further divided into 2 different groups (Tables 6 and 7).

### 3.2. Study Assessments

The following datasets were recorded for all patients: age, sex, existing diabetes or hypertension, type of surgery, anesthesia time, cardiopulmonary bypass (CPB) time, aortic cross clamp (ACC) time, use of inotropes and vasopressors, EuroSCORE, and statin therapy. Length of mechanical ventilation (LOV), stay in the ICU, and stay in the hospital were also recorded. Routine renal and liver functions were recorded. Outcome variables including acute kidney injury (AKI), postoperative atrial fibrillation (POAF), infection, stroke, wound infection, and death were noted for each patient. Data were retrieved using Dendrite Clinical Systems (London, UK). Blood samples for hsTnT and CK-MB looking for myocardial injury were collected in the first 24 h after surgery at 6 h intervals and then as per clinical indications. The hsTnT was measured using COBAS Troponin T hs (highly sensitive) STAT (short turn-around time) (Roche Diagnostics). ECG was performed routinely before and immediately after the surgery and then every 12 h. Transthoracic echocardiography was requested when indicated to trace new regional wall motion abnormalities in patients with high levels of cardiac enzymes and patients requiring high doses of inotropes.

### 3.3. Outcome Definitions

The primary outcome was the diagnosis of PMI. This was defined as a 5-fold increase in CK-MB plus one of the confirmatory criteria, including ECG, echocardiographic, or angiographic evidence. The secondary outcome measures were the length of mechanical ventilation (LOV), length of stay in the intensive care unit (LOSICU), and length of hospitalization. Based on the third universal definition of PMI (a fivefold increase in the CK-MB plus one confirmatory criterion), patients [17] were divided into two groups: no PMI (Group I) and PMI (Group II). Group II accounted for 2.17% of the study population.

### 3.4. Statistical Analysis

Normally distributed continuous variables were expressed as mean ± standard deviation (SD). Skewed variables were presented as median (interquartile range (IQR)). Patients were divided into two groups based on the existence of PMI. The groups were compared through parametric and nonparametric tests or by chi-square tests, as appropriate. Significant association was defined by a probability (*P*) value ≤ 0.05. Correlations of log hsTnT were first examined by single variable linear or logistic regression and presented as nonadjusted coefficient (NAC) and 95% confidence interval (95% CI). Analysis was done with and without adjustment of age, gender, and BMI. Factors with a *P* value ≤ 0.05 by single variable regression analyses were included in a multivariable linear regression model, presented as adjusted coefficient (AC) (95% CI) [18]. Receiver operating curve (ROC) was used to test the validity of hsTnT as a marker of PMI and to assess sensitivity and specificity. Statistical analyses were performed using the SPSS software (version 19, Chicago, IL, USA).

## 4. Results

**Figure 1:**
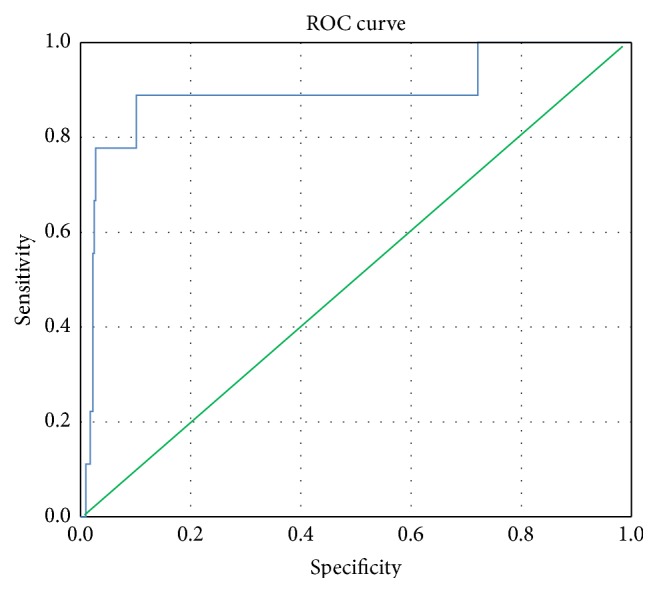
ROC for hsTnT associated definitive PMI. Receiver operating characteristic (ROC) curve for diagnostic level of highly sensitive troponin T (hsTnT) suggestive of perioperative myocardial infraction. ROC was used to discriminate hsTnT level based on CKMB cut points (above and below 120 for CKMB); 87% accuracy was detected with 95% confidence interval. Total number = 413; number of patients with definitive myocardial infarction = 9. Area under the curve (AUC) for* hsTnT *is 0.87 with *P* = 0.001.

Of the 445 patients screened, 32 were excluded; hence 413 were enrolled. The study population had a mean age of 54.9 ± 10.9 years and was predominantly male 349 (86.9%). In addition, 48.9% of the patients were diabetics and 45.6% were hypertensive. The majority of patients underwent CABG surgery (84%) (Tables 1 and 2). When patients fulfilled the diagnoses of PMI [16], ROC (Figure 1) was used to draw a corresponding level of hsTnT; a level of 3466 ng/L or above showed 90% sensitivity and 90% specificity for diagnosis of PMI with C-statistics of 0.90 (0.75–1.0). Patients with PMI had worse outcome and more complications and constituted 2.17% of the study population (Tables 3 and 4).

**Figure 2:**
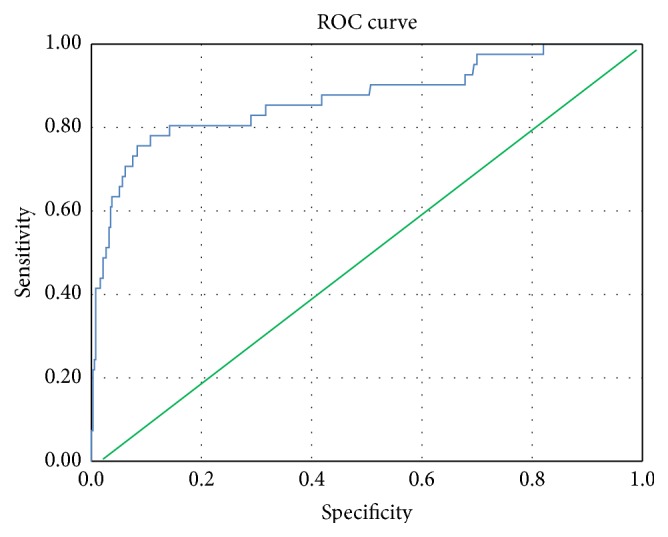
ROC for hsTnT associated high normal value of CK-MB. Receiver operating characteristic (ROC) curve for level of highly sensitive troponin T (hsTnT) suggestive of myocardial injury. ROC was used to discriminate hsTnT level based on CKMB cut points (above and below 120 for CKMB); 89% accuracy was detected with 95% confidence interval. Total number = 413; number of patients with suspicion of myocardial injury = 41. Area under the curve (AUC) for* hsTnT *is 0.89 with *P* = 0.0001. Diagonal segments are produced by ties.

We found that a hsTnT level of 2309 ng/L corresponds to the CK-MB level that is diagnostic of PMI without the additional criteria mentioned by Lurati Buse et al. [16], C-statistics of 0.87 (0.80–0.94), which showed 80% sensitivity and 86% specificity for the diagnosis of possible myocardial injury (Figure 2); therefore we conventionally considered that level as an indicator of myocardial injury and patients who were compared based on this level (Tables 5 and 6) included Groups II and IV. The two groups were well matched for age, gender, BMI, basal creatinine, association with diabetes, hypertension, and EuroSCORE. Patients with hsTnT levels of 2309 ng/L or below had a better outcome in terms of inotropes needed, length of ventilation, ICU, and hospital stay, as well as postoperative complications. We performed a multivariate analysis for significant results within the given cutoff of hsTnT (of 2309 ng/L) and found significant relations of the given level with operative emergency (*P* = 0.001); this level is a predictor for longer duration of mechanical ventilation (*P* = 0.01) and POAF (*P* = 0.003) (Table 7). This was repeated after adjustment for age, gender, and BMI, giving the same significance level.

## 5. Discussion

The salient findings of this study were the identification of similar PMI incidence as reported in other studies (2.2%) and identification of hsTnT levels that corresponds to CKMB levels indicative of myocardial injury and PMI where additional criteria were included. Finally, both levels of hsTtnT were associated with poor outcome.

In cardiac surgery settings, whether coronary artery bypass grafting or valvular surgeries, variable incidences of PMI exist, 4% in the former and up to 5% in the latter [10, 15]. In our study, the incidence of PMI for CABG and valvular surgeries were 2.3 and 1.7%, respectively. Whatever the incidence, PMI is a serious condition with a high mortality and morbidity. Thus, proper diagnostic and treating tools are needed to manage the expected high risk.

In managing patients after cardiac surgeries, early diagnosis of PMI and prediction of related morbidity are the elements that carry the greatest impact on clinical course (i.e., treatment and survival). In this context, looking for distinctive markers seems to be an ideal goal. An ideal marker should possess early diagnostic and prognostic properties. In our study, we used hsTnT, which is confirmed as offering higher sensitivity and specificity than conventional troponin (cTnI) [11].

### 5.1. Diagnostic Cutoff

We aimed to identify a diagnostic cutoff that carries high sensitivity and specificity for hsTnT, as compared to CK-MB, in the cardiac surgery setting. Thygesen and colleagues first proposed diagnostic criteria for PMI with CK-MB [17]. Lim et al. subsequently reported that the cardiac troponin I (cTnI) test at 1 h after CABG could potentially differentiate patients with significant revascularization injury; a cutoff of cTnI exceeding 5 *μ*g/L at 1 h had 67% sensitivity and 79% specificity for detecting new late gadolinium enhancement in cardiac magnetic resonance image as confirmatory [19]. We hypothesize that quantitative evaluation of hsTnT cutoff could represent a better diagnostic tool. We found that when patients fulfill diagnoses of PMI [17], ROC analysis reported that the hsTnT level of 3466 ng/L or above is associated with 90% sensitivity and 90% specificity for diagnosis of PMI (Figure 1), where the level of 2309 ng/L is associated with 80% sensitivity and 86% specificity for an equivalent level of CKMB (Figure 2) (5-fold higher than the normal level), which would be suspicious of myocardial injury (Tables 5 and 6). No previous studies have addressed a diagnostic cutoff for hsTnT.

### 5.2. Prediction of Mortality and Morbidity

Prediction of outcome in terms of short-term mortality could be achieved in noncardiac surgery through cardiac troponin I [20]. Contemporary assays should supply an appropriate diagnostic performance, where high sensitivity is a basic need. Patients' prognostication in terms of risk and possible adverse events through monitoring technique provide a great value for clinicians, allowing adjustments of preventative as well as therapeutic interventions; hsTnT is thus a marker that could help in modern clinical laboratories [21]. In our study, we hypothesize that hsTnT could provide better diagnostic and prognostic properties than conventional CKMB. This was consistent with Freund et al., who stated that higher sensitivity is attributed to hsTnT when compared with conventional cTnI in patients with low to moderate MI probability [11].

Gillies et al. pointed to the high incidence of myocardial injury after major noncardiac surgeries, where hsTnT concentration could exceed the 99th percentile in 45% of patients. The authors did not find an association between the peak postoperative troponin level and outcome [22]. The latter was contrary to Nagele et al., who found a significant association between postoperative elevation of hsTnT with MI and long-term mortality after noncardiac surgery [23]. James et al. found that base troponin provides information related to the 30-day mortality in ACS and, when it is combined with C-reactive protein, could provide a better risk identification [24].

In heart surgery, variable events could be the cause of an elevation of hsTnT, including direct surgical trauma and incomplete cardiac protection [4], end-stage renal disease [5], acute pericarditis, acute heart failure (AHF) [6], sepsis [7], and rhabdomyolysis [8]. In our study, we excluded patients with ESRD, sepsis, and preexisting high level of hsTnT.

In our study, we used equivalent levels of HsTnT to high/normal levels of CKMB to define groups. Lehrke et al., 48 h after cardiac surgery, utilized a cTnT concentration of >0.46 ng/mL to predict mortality [25]. Both groups in our work (III and IV) were matched regarding the age, sex, existence of diabetes or hypertension, BMI, EuroSCORE, basal creatinine, and EF%. The emergency of surgery did not show statistical differences, nor did the type of surgery (whether CABG or valvular). Patients with a higher level of hsTnT required more inotropes (Table 5). Similarly, Mohammed et al. found a highly significant correlation between the need for inotropic support and troponin elevation in cardiac surgery [26].

The length of the surgery did not differ between the groups; however, the lengths of CPB and ACC were significantly higher in patients with higher levels of hsTnT (Table 6). This was consistent with Järvinen et al., who performed a multivariate logistic regression analysis that concluded that long CPB is an independent predictor for PMI [27]. Length of mechanical ventilation and lengths of stay in the ICU and hospital were all significantly higher in patients with a higher level of hsTnT. Gamble et al. expressed the prognostic value in the settings of MI [28]. The incidence of complications was significantly higher in patients with a higher level of hsTnT in terms of POAF, reexploration, and mortality, where ventilator associated pneumonia (VAP) and ICU readmission were also higher but without statistical significance (Table 6). Jaffe argued that patients with high levels of hsTnT are more likely to have a troublesome course than those without similar elevation [29]. We excluded patient with preexisting high levels of hsTnT, except when the postoperative level difference was significantly high. Patients with a marked intraoperative hypotension were also excluded due to previous reports of hsTnT elevation being associated with preoperative levels [30] and intraoperative hypotension [29, 31].

Weber et al. used the additive value of the hsTnT, revised cardiac index, and N terminal probrain natriuretic peptide (NT-proBNP) to predict adverse cardiac events in major noncardiac surgeries. The authors found that hsTnT was the strongest independent risk predictor. Furthermore, the authors found that high perioperative levels of both cardiac markers were associated with the length of hospital stay and the necessity of intensive care treatment [32].

We performed multivariate analyses for the significant results within the given cutoff of hsTnT (of 2309 ng/L) and found a significant association of the given level with procedures in the emergency settings (*P* = 0.001); the level is predictor for longer duration of mechanical ventilation (*P* = 0.01) and POAF (*P* = 0.003) (Table 7). This was consistent with Beckman, who stated that prediction of all-cause mortality, as well as cardiovascular morbidity, could be achieved through troponin elevation [33]. Hernández-romero et al. similarly found that presurgical hsTnT elevation was associated with the development of POAF events, unlike the N terminal probrain natriuretic peptide (NT-proBNP) [34], whereas Laine argued that cardiac troponin may not carry prognostic information in asymptomatic patients who lack electrocardiography changes [35]. However, the study population consisted of noncardiac surgery patients.

Our results found that 15 patients (36.5% with higher level of hsTnT) (Table 6) had AKI. Evidence suggested that cardiac troponin could increase in patients with chronic renal failure even in the absence of myocardial ischemia and may add to the complexity of diagnosing ACS in this group of patients [36]. The latter authors suggested referring to a preset level of troponin when attempting to evaluate myocardial injury. Dubin et al. studied hsTnT in 81 subjects with renal failure, finding similar increased levels. However, in our study, we excluded patients with chronic renal failure [37]. Whether the rise of hsTnT in our study was a result or a cause of AKI could not be determined with certainty; however, Aviles et al. reported that, regardless of the creatinine clearance levels in patients with ACS, short-term prognosis could be predicted with cardiac troponin T levels [38].

### 5.3. Perioperative Myocardial Infarction

We found the diagnostic level of hsTnT in PMI to be 3466 ng/L or above, which is associated with 90% sensitivity and 90% specificity when including one of the confirmatory criteria, as in CK-MB [17] (Tables 3 and 4). Patients with PMI had a poorer outcome and more complications. Excellent diagnostic performance of hsTnT assays was reported, which substantially improved diagnosis of AMI in the early phase [12]. Nagele et al. highlighted the power of increased levels of hsTnT in a study including 625 patients that had undergone major noncardiac surgery, which serves for risk stratification and could be used as a tool to quantify myocardial injury in patients with cardiovascular risk factors [39]. Higher sensitivity as well as specificity due to CTnI was described when compared with CK-MB for diagnosing PMI after cardiac surgery [40], which is in line with our findings using hsTnT.

### 5.4. Strengths and Limitations

Our study sheds new light on the utilization of hsTnT as a prognostic tool, where a set level could differentiate PMI and another level was associated with morbidity and mortality after cardiac surgery. This study has the limitations of being performed at a single center, lacking a supportive measure to detect the extent of myocardial loss of viability and relates it to the used cutoff without PMI. The significant association of AKI in the high HsTNT group should be further investigated. The study population was predominantly male because the Qatari population consists of only ~20% natives, with the remaining 80% being expatriate. The majority of the latter are male.

Further studies are needed, either with large number of patients or multicenter study, to confirm our cutoff levels. Long-term follow-up studies are also needed.

## 6. Conclusion

The hsTnT levels detected here paralleled those of CK-MB and a cutoff level of 3466 ng/L could be diagnostic of PMI. Further studies are required to validate this finding. Secondary outcome measures in patients with PMI (i.e., LOV and LOS_ICU_) were significantly prolonged.

## Recommendations and Future Directions

Recommendations and future directions are as follows:

1. Utilization of hsTnT cutoff to diagnose PMI in association with other diagnostic tools.
2. Utilization of high level cutoff to prognosticate outcome after cardiac surgeries.
3. High incidence of PMI after cardiac surgeries.
4. Considering AKI when interpreting hsTnT in a justified study.

## Key Messages

Key messages are as follows:

1. Power of hsTnT to predict the outcome after cardiac surgeries.
2. Ability to have hsTnT cutoff to diagnose PMI.
3. Association of poor outcome and more complication in patients with hsTnT even without PMI.
4. Value of frequent hsTnT monitoring after cardiac surgery.
5. Value of assay of hsTnT from a reference in patients with preoperative high level.

## Figures and Tables

**Table 1:** Description of the studied group.

Variable	Number	Minimum	Maximum	Mean ± SD
Age	413	19	85	54.9 ± 10.9
BMI (kg/m^2^)	412	14.5	44.8	27.4 ± 5.1
Creatinine (micromole/L)	413	43	145	92.4 ± 53.1
EF%	413	20	65	48.6 ± 10
Additive EuroSCORE	411	0	17	3.6 ± 2.9
CPB time (minutes)	290	0	342	121.1 ± 48.1
ACC time (minutes)	286	0	215	77.2 ± 35.1
CK-MB (U/L)	413	3	737	76.9 ± 44.4
hsTnT (ng/L)	413	24	66299	1812.2 ± 111.5
Anesthesia time (minutes)	413	180	378	333.3 ± 97
LOV (minutes)	409	181	12980	566 ± 444
LOS_ICU_ (hours)	410	15	491	147 ± 68
LOS_hosp_ (days)	410	4	49	31.7 ± 29.9

BMI: body mass index; EF: ejection fraction; HgA1C: glycated hemoglobin; CPB: cardiopulmonary bypass; ACC: aortic cross clamp; hsTnT: high sensitive troponin T; LOV: length of mechanical ventilation; LOS_ICU_: length of stay in ICU; LOS_hosp_: hospital length of stay.

**Table 2:** Intergroup statistics.

Variable	Number (%)
Gender	
Male	359 (86.9)
Female	54 (13.9)

Hypertension	183 (45.6)
Diabetes	194 (48.9)

Operative urgency	
Elective	222 (53.1)
Urgent	107 (25.6)
Emergent	13 (3.1)

Surgery type	
CABG	347 (84)
Valvular surgery	60 (14.5)
Aortic dissection	6 (1.45)

Outcome	
Readmission to ICU	11 (2.6)
Reexploration	35 (14.5)
POAF	19 (4.6)
AKI	117 (28.3)
Mortality	12 (2.9)

CABG: coronary artery bypass graft; POAF: postoperative atrial fibrillation; AKI: acute kidney injury.

**Table 3:** Clinical and laboratory variables in both groups.

Variable	Group I *N* = 404 (%)	Group II (*N* = 9)	*P* value
Age	54.9 ± 10.8	55 ± 12.8	0.56

Sex, male	352 (87.1)	7 (86.4)	0.1

Hypertension	178 (44)	5 (55.5)	0.15

Diabetes	188 (46.5)	6 (66.6)	0.01

BMI	28.2 ± 5.8	32 ± 10.7	0.6

EuroSCORE	3.65 ± .5	4 ± 0.3	0.87

Basal creatinine (micromole/L)	90.5 ± 44.2	86 ± 20.4	0.21

EF%	44.7 ± 7.6	42.7 ± .1	0.4

Surgery (elective)	97 (66.4)	57 (70.4)	0.35

Inotrops			
Dopamine	30 (7.4)	7 (77.7)	0.01
Adrenaline	23 (5.7)	3 (22.2)	0.03
Noradrenline	55 (13.6)	8 (88.8)	0.01
Dobutamine	3 (0.7)	2 (33.3)	0.01

Surgery			
CABG	336 (83.3)	8 (88.8)	0.4
Valvular	65 (16.1)	1 (11.1)	
Aortic disssection	5 (1.2)	1 (11.1)	

Highest CKMB	1938.14 ± 543.1	8169.11 ± 4690.1	0.000
Highest hsTnT	54.62 ± 14.1	167.56 ± 68.387	0.000

IDDM: insulin dependent diabetes mellitus; NIDDM: non-insulin-dependent diabetes mellitus; BMI: body mass index; HbA1C: glycated hemoglobin; EF: ejection fraction; CABG: coronary artery bypass graft.

**Table 4:** Clinical outcome in both groups.

Variable	Group I (*N* = 404)	Group II (*N* = 9)	*P* value
Intraoperative parameters			
CPB time (minutes)	120.7 ± 37	134.8 ± 52.8	0.47
ACC time (minutes)	77.1 ± 34.8	77.2 ± 50.7	0.1
Anesthesia time (minutes)	6.8 ± 1.5	7 ± 1.9	0.9

Postoperative parameters			
LOV median (range) (minutes)	422 ± 211 (181–1440)	1567 ± 597 (260–129800)	0.000
LOS_ICU_ median (range) (hours)	61.6 ± 9.8 (15–320)	408.4 ± 70.5 (46–491)	0.05
LOS_hosp_ median (range) (days)	12.18 ± 2.5 (3.7–25)	18.78 ± 7.6 (5.3–49)	0.000

Postoperative complication			
POAF	14 (3.4)	5 (55.6)	0.01
AKI	120 (29)	7 (77.8)	0.04
VAP	4 (1)	2 (22.2)	
Readmision ICU	9 (2.2)	2 (22.2)	0.01
Reexploration	30 (7.4)	5 (55.6)	0.001
In-hospital mortality	9 (2.2)	3 (33.3)	0.01

CPB: cardiopulmonary bypass; ACC: aortic cross clamp; LOV: length of mechanical ventilation; LOS_ICU_: ICU length of stay; LOS_hosp_: hospital length of stay; POAF: postoperative atrial fibrillation; AKI: acute kidney injury; VAP: ventilator associated pneumonia.

**Table 5:** Clinical and laboratory variables for CKMB discrimination.

Variable	Group III CKMB < 120 *N* = 372 (%)	Group IV CKMB ≥ 120 (*N* = 41)	*P* value
Age	55.2 ± 10.6	51.7 ± 12.8	0.4

Sex, male	324 (78.8)	35 (85.3)	0.5

Hypertension	164 (44)	19 (55.5)	0.4

Diabetes	172 (44.3)	22 (66.6)	0.01

BMI	28.4 ± 5.8	28.1 ± 7.2	0.23

EuroSCORE	3.8 ± 0.4	4.1 ± 0.36	0.7

Basal creatinine (micromole/L)	90.2 ± 44.4	91.9 ± 38.4	0.6

EF%	48.8 ± 10	47.1 ± 9.1	0.8

Surgery (elective)	194 (66.4)	16 (70.4)	0.08

Inotrops			
Dopamine	27 (7.2)	10 (24.3)	0.03
Adrenaline	18 (4.8)	8 (19.5)	0.04
Noradrenline	48 (12.9)	15 (36.5)	0.01
Dobutamine	2 (0.5)	3 (7.3)	0.01

Surgery			
CABG	314 (84.4)	33 (80.4)	0.3
Valvular	53 (14.2)	7 (17)	0.3
Aortic disssection	5 (1.3)	1 (2.4)	

Highest CKMB	36.7 ± 22	242 ± 93	0.000
Highest hsTnT	1434.14 ± 150	7884 ± 190	0.000

IDDM: insulin dependent diabetes mellitus; NIDDM: non-insulin-dependent diabetes mellitus; BMI: body mass index; HbA1C: glycated hemoglobin; EF: ejection fraction; CABG: coronary artery bypass graft.

**Table 6:** Clinical outcome for CKMB discrimination.

Variable	Group III CKMB < 120 *N* = 372 (%)	Group IV CKMB ≥ 120 (*N* = 41)	*P* value
Intraoperative parameters			
CPB time (minutes)	119 ± 43	135 ± 69.6	0.06
ACC time (minutes)	77.1 ± 33	77.4 ± 48.5	0.09
Anesthesia time (minutes)	6.8 ± 1.4	6.7 ± 2	0.9

Postoperative parameters			
LOV median (range) (minutes)	402 ± 45.1 (181–1227)	767 ± 130 (198–129800)	0.001
LOS_ICU_ median (range) (hours)	57.4 ± 8.9 (15–320)	172.4 ± 37.5 (46–491)	0.000
LOS_hosp_ median (range) (days)	11 ± 3.2 (3.7–21)	15.8 ± 2 (3.3–49)	0.000

Postoperative complication			
POAF	12 (3.2)	7 (17)	0.04
AKI	102 (27.4)	15 (36.5)	0.03
VAP	4 (1)	2 (4.9)	
Readmission ICU	9 (2.4)	2 (4.9)	
Reexploration	24 (6.5)	11 (26.8)	0.05
In-hospital mortality	7 (1.9)	5 (12.1)	0.009

CPB: cardiopulmonary bypass; ACC: aortic cross clamp; LOV: length of mechanical ventilation; LOS_ICU_: ICU length of stay; LOS_hosp_: hospital length of stay; POAF: postoperative atrial fibrillation; AKI: acute kidney injury; VAP: ventilator associated pneumonia.

**Table 7:** Multivariate logistic regression analysis for hsTnT cutoff (2309 ng/L).

Variable	Adjusted OR	95% CI	*P* value
Operation emergency	10.2	2.5–41.3	0.001
LOV	1.01	1.002–1.02	0.01
AKI	0.84	0.32–2.20	0.72
POAF	4.79	1.7–13.53	0.003
Mortality	3.7	0.42–32.9	0.24

CI: confidence interval; LOV: length of ventilation; AKI: acute kidney injury; POAF: postoperative atrial fibrillation.

## Abbreviations

ACC: Aortic cross clamp
ACS: Acute coronary syndrome
AKI: Acute kidney injury
BG: Blood glucose
CABG: Coronary artery bypass graft
CAD: Coronary artery disease
CK: Creatine kinase
CK-MB: Creatine kinase MB
cTnI: Cardiac troponin I
cTnT: Cardiac troponin T
CPB: Cardiopulmonary bypass
HbA1c: Glycated hemoglobin
hsTnT: Highly sensitive troponin T
ICU: Intensive care unit
LOS: Length of stay
LOV: Length of ventilation
POAF: Postoperative atrial fibrillation
PMI: Perioperative myocardial infarction
ROC: Receiver operating curve
TnT: Cardiac troponin T.
